# l-Alanylglycylhistamine dihydro­chloride

**DOI:** 10.1107/S1600536812023562

**Published:** 2012-05-31

**Authors:** Katalin Selmeczi, Patrick Gizzi, Emmanuel Wenger, Bernard Henry

**Affiliations:** a Laboratoire SRSMC (UMR 7565 CNRS - Université de Lorraine), Groupe SUCRES, Faculté des Sciences et Technologies, BP 70239, F-54506 Vandoeuvre-lès-Nancy Cedex, France; b Laboratoire CRM2 (UMR 7036 CNRS - Université de Lorraine), Faculté des Sciences et Technologies, BP 70239, F-54506 Vandoeuvre-lès-Nancy Cedex, France

## Abstract

In the title compound {systematic name: 4-[2-({*N*-[(2*S*)-2-ammonio­propano­yl]glyc­yl}amino)­eth­yl]-1*H*-imidazol-3-ium dichloride}, C_10_H_19_N_5_O_2_^2+^·2Cl^−^, the pseudo-tripeptide l-alanyl­glycyl­histamine is protonated at both the terminal amino group and the histidine N2 atom. The resulting positive charges are neutralized by two chloride anions. In the crystal, the organic cation adopts a twisted conformation about the CH_2_—CH_2_ bond of histamine and about the C—N bond in the main chain, stabilized by a short intra­molecular C—H⋯O contact. In the crystal, N^+^—H⋯O and N^+^—H⋯Cl^−^ hydrogen bonds link the mol­ecules into infinite sheets parallel to the (100) plane. The stacking of these sheets along the *a* axis is supported by N_amide_—H⋯Cl^−^ hydrogen bonds.

## Related literature

For the complexation ability of l-Ala-Gly-HA, see: Gizzi *et al.* (2005 ▶). For bond lengths and angles in other oligopeptides, see: Itoh *et al.* (1977 ▶); Selmeczi *et al.* (2008 ▶). For discussion of hydrogen bonding, see: Steiner (2002 ▶). For the synthesis of pseudo-peptides, see: Henry *et al.* (1993 ▶). For the definition of torsion angles in peptides, see: IUPAC–IUB Commission on Biochemical Nomenclature (1970 ▶). 
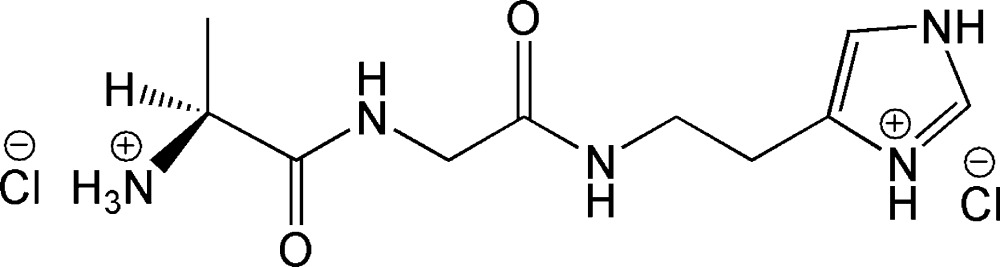


## Experimental

### 

#### Crystal data

C_10_H_19_N_5_O_2_^2+^·2Cl^−^
*M* *_r_* = 312.20
Monoclinic, 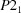

*a* = 7.5864 (3) Å
*b* = 7.4083 (3) Å
*c* = 13.7673 (6) Å
β = 105.337 (2)°
*V* = 746.20 (5) Å^3^
*Z* = 2
Mo *K*α radiation
μ = 0.44 mm^−1^
*T* = 100 K
0.45 × 0.25 × 0.11 mm

#### Data collection

Nonius KappaCCD diffractometer
15818 measured reflections
3574 independent reflections
3459 reflections with *I* > 2σ(*I*)
*R*_int_ = 0.060

#### Refinement

*R*[*F* ^2^ > 2σ(*F* ^2^)] = 0.027
*wR*(*F* ^2^) = 0.070
*S* = 1.00
3574 reflections
174 parameters
1 restraint
H-atom parameters constrained
Δρ_max_ = 0.21 e Å^−3^
Δρ_min_ = −0.23 e Å^−3^
Absolute structure: Flack (1983 ▶), with 1643 Friedel-pairs
Flack parameter: −0.03 (4)

### 

Data collection: *COLLECT* (Nonius, 1998 ▶); cell refinement: *SCALEPACK* (Otwinowski & Minor, 1997 ▶); data reduction: *DENZO* (Otwinowski & Minor, 1997 ▶) and *SCALEPACK*; program(s) used to solve structure: *SHELXS97* (Sheldrick, 2008 ▶); program(s) used to refine structure: *SHELXL97* (Sheldrick, 2008 ▶); molecular graphics: *Mercury* (Macrae *et al.*, 2008 ▶); software used to prepare material for publication: *enCIFer* (Allen *et al.*, 2004 ▶).

## Supplementary Material

Crystal structure: contains datablock(s) I, global. DOI: 10.1107/S1600536812023562/fy2052sup1.cif

Structure factors: contains datablock(s) I. DOI: 10.1107/S1600536812023562/fy2052Isup2.hkl

Supplementary material file. DOI: 10.1107/S1600536812023562/fy2052Isup3.cml

Additional supplementary materials:  crystallographic information; 3D view; checkCIF report

## Figures and Tables

**Table 1:** Hydrogen-bond geometry (Å, °)

*D*—H⋯*A*	*D*—H	H⋯*A*	*D*⋯*A*	*D*—H⋯*A*
N1—H1*N*⋯Cl2	0.88	2.21	3.0503 (15)	159
N2—H2*N*⋯O1^i^	0.88	1.82	2.6962 (19)	175
N4—H4⋯Cl1^ii^	0.88	2.48	3.3100 (13)	157
N5—H5*A*⋯Cl1	0.91	2.38	3.2046 (14)	151
N5—H5*B*⋯Cl2^iii^	0.91	2.24	3.1130 (14)	161
N5—H5*C*⋯Cl1^iv^	0.91	2.37	3.2676 (14)	170
C3—H3⋯O2	0.95	2.30	3.2074 (19)	161

Symmetry codes: (i) 
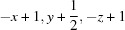
; (ii) 
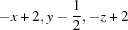
; (iii) 
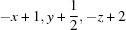
; (iv) 
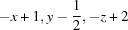
.

**Fig. 1.**
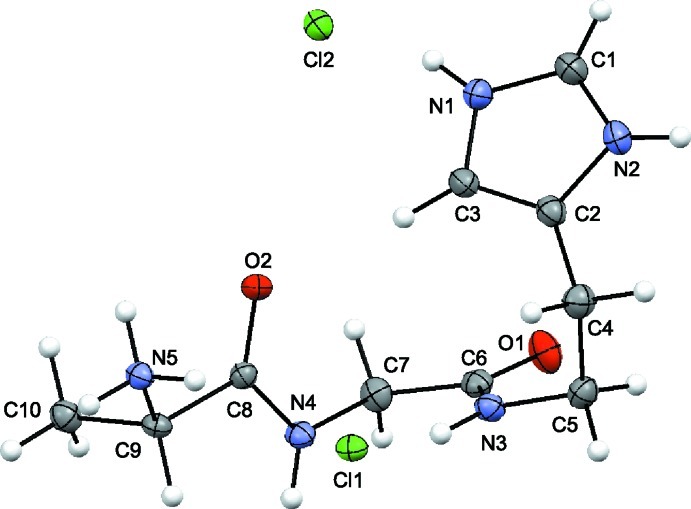
Molecular structure of L-Ala-Gly-HA.2HCl with the atom-numbering scheme. Displacement ellipsoids are drawn at the 50% probability level.

**Fig. 2.**
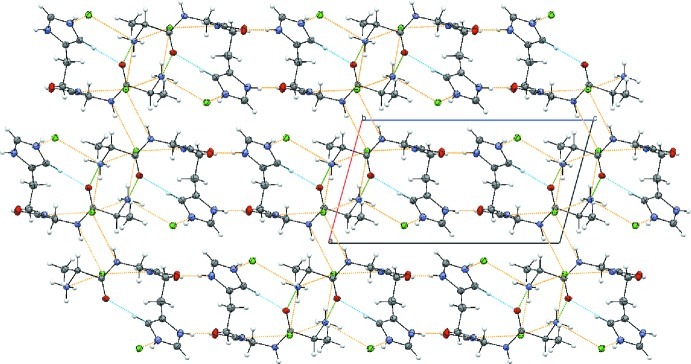
A view of the crystal packing of the title compound along the b axis, showing the N—H···O and N—H···Cl (orange dotted line), C—H···O (light blue dotted line) hydrogen bonds and N—O short contacts (green dotted line) in the (100) plane.

## supplementary crystallographic information

### Comment

The metal complexation ability of the N-terminal sequence in serum albumin (HSA, involved in the transport of metal ions in blood) is among the best studied examples of peptide–metal interactions. The so-called ATCUN-motif of HSA (Amino Terminal Cu(II) and Ni(II) binding site) was mimicked, among others, by the ligand *L*-Alanyl-Glycyl Histamine (*L*-Ala-Gly-HA) (Gizzi *et al.*, 2005). We report here the molecular structure of the dihydrochloride salt of the pseudo-tripeptide *L*-Ala-Gly-HA. In the title compound, the *L*-Ala-Gly-HA part is doubly positively charged on the amino and the imidazole groups. This double charge is neutralized by the presence of two chloride ions (Fig. 1). The absolute configuration (S) of atom C9 was assumed from the stereochemistry of the precursor Boc-*L*-Ala-Gly-OH. In the organic cation the bond distances and angles of the peptide bonds and of the protonated imidazole ring are close to the values measured for other oligopeptides (Itoh *et al.*, 1977; Selmeczi *et al.*, 2008). The conformation of the title tripeptide can be determined by analysis of the torsion angles about the C—C (ψ), C—N (ω) and N—C (φ) bonds (IUPAC–IUB Commission on Biochemical Nomenclature, 1970). The conformation may be considered as fully extended (open) if the magnitude of ψ, ω and φ angles is near 180° (e.g. for Gly-β-Ala-Histamine; Selmeczi *et al.*, 2008). The torsion angle ψ about the C4—C5 bond in *L*-Ala-Gly-HA is -56.01° resulting in the folding back of the imidazole ring on the N3—C6—O1 bonds. In addition, the main chain of the tripeptide adopts a twisted conformation defined by the small value of torsion angle of φ about N4—C7 (90.63°) and of ψ about C7—C6 (-1.62°). These torsion angle values show the folded (closed) conformation of *L*-Ala-Gly-HA. All protons attached to the N1, N2, N4 and N5 nitrogen atoms are involved in moderate hydrogen bonding (Steiner, 2002). The N1 and N5 nitrogen atoms form N—H···Cl hydrogen bonds with the Cl2 and Cl1, Cl2^iii^, Cl1^iv^ atoms, respectively [symmetry codes: (iii) -*x* + 1, *y* + 1/2, -*z* + 2, (iv) -*x* + 1, *y* - 1/2, -*z* + 2]. The N2 nitrogen atom forms stronger H-bond with the O1^i^ carbonyl oxygen atom of a neighbouring peptide molecule [symmetry code: (i) -*x* + 1, *y* + 1/2, -*z* + 1]. The C3—H···O2 intramolecular contact is also included in the H-bond list. This interaction results from the bending back of imidazole ring on the peptide main chain and it stabilizes the `closed' conformation of the molecule. The O2 carbonyl oxygen atom participates in relatively close interaction with the neighbouring N5^iii^ nitrogen atom, reflecting a partial positive charge on the latter (distance O2···N5 is 2.917 Å). These hydrogen bonds link the molecules into infinite two-dimensional sheets parallel to the (100) plane forming a stacking structure along the *a* axis (Fig. 2). These horizontal layers are interlinked by an another N—H···Cl hydrogen bond present in the structure between N4 and Cl1^ii^ [symmetry code: (ii) -*x* + 2, *y* - 1/2, -*z* + 2], thus forming a three-dimensional framework.

### Experimental

The title compound was synthesized in one step according to the procedure described earlier (Henry *et al.*, 1993). Histamine dihydrochloride and ethyl-diisopropyl-amine in chloroform were added to the commercially available *N*-(*tert*-butoxycarbonyl)-*L*-alanyl-glycine-OH (Boc-*L*-Ala-Gly-OH) at room temperature. The mixture was stirred for 10 additional hours at rt. Deprotection of the primary amine was performed with a mixture of HCl/Et_2_O. The title compound was obtained as white powder with 60% of yield. Suitable crystals were obtained by slow evaporation of water from an acidic aqueous solution (pH 2) of the title compound. ESI-MS^+^ (*m/z*): calculated for C_10_H_17_N_5_O_2_ 239.14, found 240.20. Anal. calc. for C_10_H_17_N_5_O_2_.2HCl: C, 38.47; H, 6.13; N, 22.43. Found: C, 37.96; H, 6.08; N, 21.87%.

### Refinement

The absolute configurations of the title compound was known from the method of synthesis (enantiomer S) and it was also confirmed from the diffraction experiments. All H atoms were located in difference Fourier maps. The C/N-bonded H atoms were placed at calculated positions and refined using a riding model, with C_methyl_—H distance of 0.98 Å, C_methylene_—H distance of 0.99 Å, C_methine_—H distance of 1 Å, C_aryl_—H distance of 0.95 Å, and with N—H distance of 0.88 Å. The H-atom *U*_iso_ parameters were fixed at 1.2*U*_eq_(C) for methine, methylene and aryl C—H, at 1.5*U*_eq_(C) for methyl C—H, at 1.2*U*_eq_(C) for aryl C—H and at 1.2*U*_eq_(N) for the N—H group.

### Crystal data

**Table:**

C_10_H_19_N_5_O_2_^2+^·2Cl^−^	*F*(000) = 328
*M**_r_* = 312.20	*D*_x_ = 1.390 Mg m^−^^3^
Monoclinic, *P*2_1_	Mo *K*α radiation, λ = 0.71073 Å
Hall symbol: P2yb	Cell parameters from 1776 reflections
*a* = 7.5864 (3) Å	θ = 0.4–30.0°
*b* = 7.4083 (3) Å	µ = 0.44 mm^−^^1^
*c* = 13.7673 (6) Å	*T* = 100 K
β = 105.337 (2)°	Prismatic, colourless
*V* = 746.20 (5) Å^3^	0.45 × 0.25 × 0.11 mm
*Z* = 2	

### Data collection

**Table:**

Nonius KappaCCD diffractometer	3459 reflections with *I* > 2σ(*I*)
Radiation source: fine-focus sealed tube	*R*_int_ = 0.060
Horizonally mounted graphite crystal monochromator	θ_max_ = 28.0°, θ_min_ = 2.8°
Detector resolution: 9 pixels mm^-1^	*h* = −10→10
ω scans	*k* = −9→9
15818 measured reflections	*l* = −18→18
3574 independent reflections	

### Refinement

**Table:**

Refinement on *F*^2^	Secondary atom site location: difference Fourier map
Least-squares matrix: full	Hydrogen site location: inferred from neighbouring sites
*R*[*F*^2^ > 2σ(*F*^2^)] = 0.027	H-atom parameters constrained
*wR*(*F*^2^) = 0.070	*w* = 1/[σ^2^(*F*_o_^2^) + (0.0395*P*)^2^ + 0.2259*P*] where *P* = (*F*_o_^2^ + 2*F*_c_^2^)/3
*S* = 1.00	(Δ/σ)_max_ = 0.001
3574 reflections	Δρ_max_ = 0.21 e Å^−^^3^
174 parameters	Δρ_min_ = −0.23 e Å^−^^3^
1 restraint	Absolute structure: Flack (1983), with 1643 Friedel-pairs
Primary atom site location: structure-invariant direct methods	Flack parameter: −0.03 (4)

### Special details

**Table:**

Geometry. All e.s.d.'s (except the e.s.d. in the dihedral angle between two l.s. planes) are estimated using the full covariance matrix. The cell e.s.d.'s are taken into account individually in the estimation of e.s.d.'s in distances, angles and torsion angles; correlations between e.s.d.'s in cell parameters are only used when they are defined by crystal symmetry. An approximate (isotropic) treatment of cell e.s.d.'s is used for estimating e.s.d.'s involving l.s. planes.

### Fractional atomic coordinates and isotropic or equivalent isotropic displacement parameters (Å^2^)

**Table:**

	*x*	*y*	*z*	*U*_iso_*/*U*_eq_	
Cl1	0.75003 (4)	0.96855 (5)	0.94053 (3)	0.01717 (9)	
Cl2	0.14366 (5)	0.19501 (5)	0.69324 (3)	0.01977 (9)	
O2	0.54905 (14)	0.49471 (17)	0.89693 (8)	0.0181 (2)	
O1	0.73519 (18)	0.52373 (18)	0.61858 (9)	0.0267 (3)	
N3	0.76898 (17)	0.74018 (19)	0.73704 (10)	0.0178 (3)	
H3N	0.8002	0.7654	0.8018	0.021*	
N5	0.64458 (17)	0.66369 (18)	1.08050 (10)	0.0161 (3)	
H5A	0.6377	0.7686	1.0453	0.024*	
H5B	0.6819	0.6876	1.1477	0.024*	
H5C	0.5326	0.6103	1.0657	0.024*	
N4	0.84631 (16)	0.4832 (2)	0.89235 (9)	0.0169 (3)	
H4	0.9605	0.5056	0.9251	0.020*	
N1	0.21053 (19)	0.5728 (2)	0.62237 (11)	0.0213 (3)	
H1N	0.1619	0.4744	0.6401	0.026*	
N2	0.2662 (2)	0.7937 (2)	0.53338 (11)	0.0213 (3)	
H2N	0.2601	0.8665	0.4821	0.026*	
C7	0.8047 (2)	0.4224 (2)	0.78845 (13)	0.0196 (3)	
H7A	0.6967	0.3421	0.7760	0.023*	
H7B	0.9087	0.3489	0.7801	0.023*	
C2	0.3860 (2)	0.8128 (2)	0.62861 (12)	0.0192 (3)	
C8	0.7130 (2)	0.5057 (2)	0.93940 (11)	0.0156 (3)	
C9	0.7782 (2)	0.5401 (2)	1.05237 (11)	0.0152 (3)	
H9	0.9015	0.5982	1.0689	0.018*	
C4	0.5264 (2)	0.9575 (2)	0.65566 (13)	0.0222 (3)	
H4A	0.5219	1.0119	0.7207	0.027*	
H4B	0.4969	1.0534	0.6038	0.027*	
C3	0.3491 (2)	0.6734 (2)	0.68410 (12)	0.0195 (3)	
H3	0.4077	0.6494	0.7528	0.023*	
C6	0.7665 (2)	0.5683 (2)	0.70817 (12)	0.0189 (3)	
C10	0.7887 (2)	0.3630 (2)	1.10983 (12)	0.0198 (3)	
H10A	0.8346	0.3861	1.1822	0.030*	
H10B	0.8716	0.2799	1.0883	0.030*	
H10C	0.6666	0.3090	1.0959	0.030*	
C5	0.7215 (2)	0.8883 (2)	0.66454 (13)	0.0206 (3)	
H5D	0.7305	0.8461	0.5978	0.025*	
H5E	0.8095	0.9884	0.6862	0.025*	
C1	0.1628 (2)	0.6491 (2)	0.53191 (13)	0.0225 (3)	
H1	0.0702	0.6071	0.4757	0.027*	

### Atomic displacement parameters (Å^2^)

**Table:**

	*U*^11^	*U*^22^	*U*^33^	*U*^12^	*U*^13^	*U*^23^
Cl1	0.01378 (15)	0.01813 (17)	0.01988 (17)	−0.00020 (13)	0.00496 (12)	−0.00123 (13)
Cl2	0.01907 (17)	0.02083 (19)	0.01823 (17)	−0.00004 (13)	0.00283 (13)	0.00210 (13)
O2	0.0129 (5)	0.0223 (6)	0.0183 (5)	−0.0021 (4)	0.0025 (4)	−0.0001 (5)
O1	0.0369 (7)	0.0261 (7)	0.0169 (5)	0.0044 (5)	0.0070 (5)	−0.0050 (5)
N3	0.0164 (6)	0.0187 (6)	0.0176 (6)	−0.0024 (5)	0.0033 (5)	−0.0018 (5)
N5	0.0142 (6)	0.0174 (7)	0.0165 (6)	0.0004 (5)	0.0036 (5)	−0.0007 (5)
N4	0.0134 (5)	0.0210 (7)	0.0165 (6)	0.0013 (5)	0.0042 (4)	−0.0017 (5)
N1	0.0191 (6)	0.0214 (7)	0.0222 (7)	−0.0018 (5)	0.0036 (5)	0.0044 (6)
N2	0.0220 (7)	0.0233 (7)	0.0178 (6)	0.0015 (5)	0.0038 (5)	0.0049 (5)
C7	0.0218 (7)	0.0188 (8)	0.0189 (7)	0.0021 (6)	0.0068 (6)	−0.0036 (6)
C2	0.0173 (7)	0.0207 (8)	0.0191 (7)	0.0030 (6)	0.0040 (6)	0.0013 (6)
C8	0.0150 (6)	0.0142 (7)	0.0174 (7)	−0.0007 (5)	0.0040 (5)	0.0004 (5)
C9	0.0122 (6)	0.0158 (7)	0.0172 (7)	0.0017 (5)	0.0032 (5)	−0.0014 (5)
C4	0.0226 (7)	0.0175 (7)	0.0262 (8)	−0.0001 (7)	0.0058 (6)	0.0016 (7)
C3	0.0176 (7)	0.0219 (8)	0.0182 (7)	0.0001 (6)	0.0034 (6)	0.0018 (6)
C6	0.0150 (7)	0.0223 (8)	0.0201 (7)	0.0007 (6)	0.0061 (6)	−0.0015 (6)
C10	0.0209 (7)	0.0173 (8)	0.0202 (7)	0.0009 (6)	0.0037 (6)	0.0007 (6)
C5	0.0210 (7)	0.0184 (8)	0.0231 (8)	−0.0017 (6)	0.0072 (6)	0.0016 (6)
C1	0.0205 (8)	0.0262 (9)	0.0189 (8)	−0.0010 (6)	0.0018 (6)	0.0019 (6)

### Geometric parameters (Å, º)

**Table:**

O2—C8	1.2292 (18)	C7—C6	1.518 (2)
O1—C6	1.238 (2)	C7—H7A	0.9900
N3—C6	1.333 (2)	C7—H7B	0.9900
N3—C5	1.463 (2)	C2—C3	1.357 (2)
N3—H3N	0.8800	C2—C4	1.488 (2)
N5—C9	1.491 (2)	C8—C9	1.524 (2)
N5—H5A	0.9100	C9—C10	1.523 (2)
N5—H5B	0.9100	C9—H9	1.0000
N5—H5C	0.9100	C4—C5	1.540 (2)
N4—C8	1.3478 (19)	C4—H4A	0.9900
N4—C7	1.452 (2)	C4—H4B	0.9900
N4—H4	0.8800	C3—H3	0.9500
N1—C1	1.328 (2)	C10—H10A	0.9800
N1—C3	1.382 (2)	C10—H10B	0.9800
N1—H1N	0.8800	C10—H10C	0.9800
N2—C1	1.325 (2)	C5—H5D	0.9900
N2—C2	1.391 (2)	C5—H5E	0.9900
N2—H2N	0.8800	C1—H1	0.9500
			
C6—N3—C5	122.12 (14)	C8—C9—C10	110.15 (13)
C6—N3—H3N	118.9	N5—C9—H9	109.6
C5—N3—H3N	118.9	C8—C9—H9	109.6
C9—N5—H5A	109.5	C10—C9—H9	109.6
C9—N5—H5B	109.5	C2—C4—C5	112.89 (14)
H5A—N5—H5B	109.5	C2—C4—H4A	109.0
C9—N5—H5C	109.5	C5—C4—H4A	109.0
H5A—N5—H5C	109.5	C2—C4—H4B	109.0
H5B—N5—H5C	109.5	C5—C4—H4B	109.0
C8—N4—C7	121.08 (13)	H4A—C4—H4B	107.8
C8—N4—H4	119.5	C2—C3—N1	107.51 (14)
C7—N4—H4	119.5	C2—C3—H3	126.2
C1—N1—C3	108.74 (15)	N1—C3—H3	126.2
C1—N1—H1N	125.6	O1—C6—N3	122.41 (16)
C3—N1—H1N	125.6	O1—C6—C7	119.02 (15)
C1—N2—C2	109.45 (14)	N3—C6—C7	118.57 (14)
C1—N2—H2N	125.3	C9—C10—H10A	109.5
C2—N2—H2N	125.3	C9—C10—H10B	109.5
N4—C7—C6	116.51 (14)	H10A—C10—H10B	109.5
N4—C7—H7A	108.2	C9—C10—H10C	109.5
C6—C7—H7A	108.2	H10A—C10—H10C	109.5
N4—C7—H7B	108.2	H10B—C10—H10C	109.5
C6—C7—H7B	108.2	N3—C5—C4	111.23 (14)
H7A—C7—H7B	107.3	N3—C5—H5D	109.4
C3—C2—N2	105.84 (15)	C4—C5—H5D	109.4
C3—C2—C4	130.41 (15)	N3—C5—H5E	109.4
N2—C2—C4	123.71 (15)	C4—C5—H5E	109.4
O2—C8—N4	123.82 (14)	H5D—C5—H5E	108.0
O2—C8—C9	120.74 (14)	N2—C1—N1	108.46 (15)
N4—C8—C9	115.40 (13)	N2—C1—H1	125.8
N5—C9—C8	107.95 (12)	N1—C1—H1	125.8
N5—C9—C10	109.86 (13)		
			
C8—N4—C7—C6	90.63 (19)	N2—C2—C3—N1	0.45 (19)
C1—N2—C2—C3	−0.2 (2)	C4—C2—C3—N1	−177.31 (17)
C1—N2—C2—C4	177.72 (16)	C1—N1—C3—C2	−0.5 (2)
C7—N4—C8—O2	−7.3 (2)	C5—N3—C6—O1	4.6 (2)
C7—N4—C8—C9	170.48 (14)	C5—N3—C6—C7	−175.71 (14)
O2—C8—C9—N5	−35.58 (19)	N4—C7—C6—O1	178.07 (14)
N4—C8—C9—N5	146.53 (14)	N4—C7—C6—N3	−1.6 (2)
O2—C8—C9—C10	84.37 (18)	C6—N3—C5—C4	102.05 (17)
N4—C8—C9—C10	−93.53 (16)	C2—C4—C5—N3	−56.02 (19)
C3—C2—C4—C5	72.3 (2)	C2—N2—C1—N1	−0.1 (2)
N2—C2—C4—C5	−105.10 (18)	C3—N1—C1—N2	0.4 (2)

### Hydrogen-bond geometry (Å, º)

**Table:**

*D*—H···*A*	*D*—H	H···*A*	*D*···*A*	*D*—H···*A*
N1—H1*N*···Cl2	0.88	2.21	3.0503 (15)	159
N2—H2*N*···O1^i^	0.88	1.82	2.6962 (19)	175
N4—H4···Cl1^ii^	0.88	2.48	3.3100 (13)	157
N5—H5*A*···Cl1	0.91	2.38	3.2046 (14)	151
N5—H5*B*···Cl2^iii^	0.91	2.24	3.1130 (14)	161
N5—H5*C*···Cl1^iv^	0.91	2.37	3.2676 (14)	170
C3—H3···O2	0.95	2.30	3.2074 (19)	161

Symmetry codes: (i) −*x*+1, *y*+1/2, −*z*+1; (ii) −*x*+2, *y*−1/2, −*z*+2; (iii) −*x*+1, *y*+1/2, −*z*+2; (iv) −*x*+1, *y*−1/2, −*z*+2.
